# Perspectives on the complex links between depression and dementia

**DOI:** 10.3389/fnagi.2022.821866

**Published:** 2022-08-24

**Authors:** Antoine Hakim

**Affiliations:** ^1^Brain and Mind Research Institute, University of Ottawa, Ottawa, ON, Canada; ^2^Division of Neurology, University of Ottawa, Ottawa, ON, Canada

**Keywords:** depression, dementia, inflammation, cerebral small vessel disease, treatment, risk factors

## Abstract

This review highlights that depression is a growing health problem for the individual, and because of its high frequency in most societies, a growing burden on health care budgets. The focus of the review is the physiological links between depression and dementia, specifically Alzheimer’s disease. It suggests that depression is a significant risk factor for cognitive decline and explores the pathways that may lead depressed individuals to suffer this outcome. This review shows that depression and a number of its precursors activate pro-inflammatory mediators. These lead to cerebral small vessel disease with the consequent reduction in cerebral blood flow, which is known to precede cognitive decline. Thus, the impact of depression on the physiological events that lead to dementia is identical to the impact of other dementia risk factors recently reviewed. Depression is distinct, however, in being a relatively treatable condition, but the impact of treating depression on later cognitive decline is not always positive, leading to the hypothesis that only the antidepressants that attenuate inflammation alleviate subsequent cognitive decline.

## Introduction

Caring for dementia patients is consuming growing portions of the health care budgets of many countries. The recognition that certain risk factors increase the risk for dementia provides the possibility of reducing this burden by intensifying efforts to reduce and control the risk factors. In a recent article (Hakim, 2021), a hypothesis was presented that a sequence of physiological events links dementia risk factors to their cognitive outcomes. It was proposed that in the presence of a risk factor, the sequence that leads to dementia is triggered by inflammation which leads to cerebral small vessel disease. This results in a reduction of cerebral perfusion, which precedes the appearance of any clinical evidence for cognitive impairment. Data were presented to highlight this sequence in three recognized dementia risk factors: obesity, sedentary lifestyle, and insufficient sleep. Depression is recognized as a risk factor for dementia, and the current literature review explores the pathway from depression to dementia and suggests that it follows the same sequence of physiological events that link other risk factors to dementia. A significant difference between depression and the other risk factors is the possibility that some antidepressant medications may alleviate this risk to cognitive decline. This benefit, however, may be limited to the antidepressants that reduce inflammation.

## Dementia and depression are growing problems

All published estimates agree that the number of people affected by dementia will substantially increase over time, mainly due to projected trends in population aging and growth. The GBD2019 Dementia Forecasting Collaborators estimate the number of people with dementia globally will increase from 57.4 million in 2019 to 152.8 million in 2050 (GBD 2019 Dementia Forecasting Collaborators, 2022). Wittenberg et al. (2020) in 2020 projected that the number of older people with dementia will more than double in the next 25 years.

When dementia is estimated by age group, a clear correlation is evident between aging and prevalence of the condition. Gautrin et al. (1990) calculated dementia prevalence to be 1% for ages 65–74, 4% for ages 75–84, and 10.5% for 85 and over. In contrast, depression is not more prevalent with age even though its prevalence is also rising in many jurisdictions. A community-based study of American adults found that the 1-year frequency of major depressive disorder (MDD) rose from 3.33 to 7.06% between 1991–92 and 2001–02 (Compton, 2006). An analysis of the Minnesota Multiphasic Personality Inventory (MMPI), which consists of data on 63,706 American college students and 13,870 high school students revealed that younger adults were 6–8 times more likely than older adults to meet the criteria for clinical depression in 2007 compared to peers in 1938 (Twenge et al., 2010). In a Swedish population studied approximately every 10 years, it was shown that the risk for depression in young adults had increased 10-fold from 1957 to 1972 compared to the period from 1947 to 1957 (Hagnell, 1989; Hagnell et al., 1993). A data brief from the US department of Health and Human Services reported in September 2020 that the percentage of adults who experienced any symptoms of depression was highest among those aged 18–29 (21.0%), followed by those aged 45–64 and those older than 65 (18.4%), and lastly by those aged 30–44 (16.8%). For all degrees of depression severity, women were more likely to be affected than men (Villarroel and Terlizzi, 2020). There is thus a concordance of studies showing that the incidence of depression is rising and that the younger adult age groups are more likely to develop depression, with onset at increasingly earlier ages (Hidaka, 2012). We may therefore be in the middle of an epidemic of depression, and its impact on cognitive functions is worthy of further analysis.

## Depression increases the risk for dementia

Cognitive decline in later life has been associated with many factors; a review of these factors revealed a link between depression and the onset of dementia (Steffens et al., 2004). In a 14-year longitudinal study which followed 4,922 healthy men aged 71–89 years, 18.3% developed dementia. Interestingly, the men who were older and had a history of depression were at greater risk of developing dementia (Almeida et al., 2017). The authors concluded that the link between depression and cognitive decline was evident during the initial 5 years of follow-up. Zeki Al Hazzouri et al. (2018) reported in the Northern Manhattan Study (2018) that greater depressive symptoms, adjusted for other variables, were significantly associated with worse baseline episodic memory in populations. Greater depressive symptoms were significantly related to poorer baseline episodic memory function (β [95% confidence interval] = −0.21 [−0.33 to −0.10],*p* = 0.0003) even when the models had been adjusted for socio-demographics, vascular risk factors, and medications for behavioral and mental health issues (Zeki Al Hazzouri et al., 2018). Gatchel et al. (2019), in a longitudinal study of 276 cognitively unimpaired older adults, showed that worsening depressive symptoms were significantly associated with declining cognition. Finally, researchers have concluded that half of the patients with major depressive disorders showed cognitive and memory impairments (Köhler et al., 2010). Norton et al. (2014) predicted that depression accounted for 5–11% of all Alzheimer’s disease cases. There is therefore concordant evidence in the literature that depression is a significant risk factor for cognitive decline.

## The risk factors for depression

It is now widely appreciated that depression can have a number of precursors and social determinants (Slavich and Irwin, 2014). Exposure to early life stressors such as social stressors, social isolation, and the inability to form attachments are all possible risk factors for depression (Panksepp, 2003; Watt and Panksepp, 2009). Early maltreatment has been associated with late-life depression and suicide risk (Comijs et al., 2013). Watt and Panksepp (2009) conceptualize depression as arising from an evolutionarily preserved “shutdown mechanism” resulting from protracted separation distress in early life. These types of stressors have been linked to inflammation and changes in immune function which may lead to both depression and dementia.

In 2003, Eisenberger et al. (2003), demonstrated that with increasing social distress greater activity could be observed in the anterior cingulate cortex, an area implicated in generating the aversive experience of physical pain. A study done by Slavich and Irwin (2014), examined young adults who were exposed to social stressors while monitoring markers of inflammatory activity and brain activity using fMRI. Social stress exposure resulted in significant increases in a soluble receptor for tumor necrosis factor alpha and interleukin-6. The TNF-alpha receptor increases were associated with greater activity in the dorsal anterior cingulate cortex and anterior insula. These regions have been previously associated with processing rejection-related distress. A second study by Muscatell et al. (2015) showed that higher levels of neuronal activity in the amygdala in response to stress was associated with greater increases in inflammation.

Overall, these studies suggest that proinflammatory cytokines are the crucial mediators between the risk factors and their depression consequences often evident as sad mood, chronic feeling of fatigue, social withdrawal, and anhedonia. Therefore, targeting inflammation may offer new opportunities for preventing and treating depression.

## The pathogenesis of cognitive decline. Alzheimer’s disease is the major contributor to dementia

Crous-Bou et al. (2017) state that Alzheimer’s disease accounts for 60–80% of dementias, making it the most common precursor of cognitive decline. In that condition the hallmark pathological criteria have included elevated levels of amyloid-beta peptide and hyperphosphorylated tau which accumulates intracellularly and becomes microscopically evident as neurofibrillary tangles. Recent evidence, however, suggests that a decline in cerebral blood flow precedes these pathological hallmarks of Alzheimer’s disease, potentially by many years. In an extensive study by Iturria-Medina et al. (2016), where multiple simultaneous measurements of regional cerebral perfusion and other biomarkers of Alzheimer’s disease were made, a decline in cerebral perfusion preceded all other pathological hallmarks of Alzheimer’s disease. Bangen et al. (2018) have subsequently confirmed that reduced regional cerebral blood flow relates to poorer cognition in adults with type 2 diabetes. More recently, Bracko et al. (2021) confirmed the crucial role that a reduction in cerebral blood flow plays in Alzheimer’s disease.

## The pathways from depression to dementia

### Depression is associated with a reduction in cerebral blood flow

Multiple studies have shown that cerebral blood flow is reduced in the setting of depression. Popa-Wagner et al. (2015) eloquently described in 2015 how dysfunction of cerebral autoregulation in aging can impair CBF and increase susceptibility to hypoxia and ischemia. Using arterial spin labeling, Cooper et al. (2020) compared cerebral blood flow (CBF) between 164 individuals suffering from major depression and 94 healthy controls. They reported reduced CBF in the right parahippocampus, thalamus, fusiform, and middle temporal gyri along with bilateral insula regions in depressed patients compared to controls. This confirmed the results obtained by Meyer et al. (1973) who showed that in severe depression there is bilateral hemispheric reduction of CBF. Takano et al. (2006) reported that the regional CBF (rCBF) in depressed patients was decreased compared to normal controls in widespread areas including the frontal lobe and limbic regions such as the cingulate cortex and parahippocampal gyrus. Oda et al. (2003) showed in depressed patients that rCBF in the frontal lobe, temporal lobe, and anterior cingulate gyrus were reduced regardless of the presence of subcortical hyperintensities. Where there was MRI hyperintensity, however, patients displayed reduced rCBF in the thalamus, basal ganglia, and brainstem a long with the cortical areas. In addition, the white matter hyperintensity scale was negatively correlated with rCBF in subcortical brain regions, such as the thalamus and right basal ganglia (Oda et al., 2003).

Depression also modifies vascular risk factors (Hakim, 2011). The effect of emotions on heart and blood vessel function was investigated. It was found that sadness created a distinctive pattern, showing slight increases in blood pressure and vascular resistance, and a reduction in the pumping capacity of the heart (Sinha et al., 1992). Thus, the reduction in CBF seen in depressed individuals may partially be the result of the impact of sadness on vascular risk factors.

### Cerebral small vessel disease is evident in depressed individuals

Depression is as immense a risk factor for small vessel disease as high blood pressure (Wang et al., 2014). People who are depressed have abnormalities in the same brain regions known to be at risk for the development of small covert white matter strokes (Aizenstein et al., 2011). Greater depressive symptoms, after adjusting for sociodemographic, behavioral, and vascular risk factor variables, are correlated with smaller cerebral parenchymal fraction (β [95% confidence interval] = −0.56 [−1.05 to −0.07], *p* = 0.02) and increased odds of subclinical brain infarcts (odds ratio [95% confidence interval] = 1.55 [1.00–2.42], *p* = 0.05) (Zeki Al Hazzouri et al., 2018). Although this publication concludes that increased symptoms of depression were not significantly linked to white matter hyper-intensity volume, numerous magnetic resonance imaging (MRI) investigations have demonstrated that late life depression is related to the increased prevalence of white matter hyper-intensities on MRI (Krishnan et al., 2004; Taylor et al., 2005, 2013; Chen et al., 2006; Herrmann et al., 2008; Firbank et al., 2012).

### The role of inflammation in depression

Possibly the most important mechanism associating depression with cognitive decline is how the immune system responds to persistent depression. This topic was extensively covered recently in the excellent article by Dafsari and Jessen (2020), where it was concluded that depressed patients exhibit chronic inflammation. Elevations in the interleukin system and tissue necrosis factor (TNFα) and C-reactive protein (CRP) have been reported in depressed patients, frequently associated with a simultaneous decrease in anti-inflammatory regulation (Dowlati et al., 2010; Felger and Lotrich, 2013). Miller and Raison (2016) concluded from meta-analyses that the most consistent biomarkers of inflammation in patients with depression were the peripheral blood interleukins, such as interleukin (IL)-1β, IL-6, TNFα, and CRP. Polymorphisms in inflammatory cytokine genes have been linked to depression and the individual’s response to therapy. These polymorphisms are for genes such as IL-Iβ, TNFα, and CRP (Bufalino et al., 2013). Other genes that have been linked to depression come from meta-analyses of genome-wide association studies and are associated with the immune system’s response to pathogens (Raison and Miller, 2013). It has been shown that if non-depressed persons are given inflammatory cytokines such as IFNα the onset of signs of depression occurs (Reichenberg et al., 2001; Bonaccorso et al., 2002; Capuron et al., 2002; Harrison et al., 2009). In addition, if cytokines like TNFα, or components of the inflammatory signaling pathway like cyclooxygenase 2, can be reduced, then the signs of depression can be reduced in individuals with various medical conditions such as rheumatoid arthritis, psoriasis and cancer, and major depressive disorder (Tyring et al., 2006; Köhler et al., 2014; Abbott et al., 2015). These findings highlight that depression has a large influence on the inflammatory pathways.

## The impacts of treating depression

Depression can be managed by a variety of means, including psychotherapy, electroconvulsive therapy, and anti-depressant medication. This review will focus on the more prevalent therapy, namely oral antidepressants.

There is considerable debate on the impact of treating depression on the subsequent development of dementia. Coupland et al. (2019) presented evidence from a case-control study in which they reported that anticholinergic antidepressants such as Paxil and other tricyclic antidepressants may actually increase rather than decrease the risk of subsequent Alzheimer’s disease. Similar negative impact on cognition is attributed to SSRI’s (Wang et al., 2016). Consequently, when faced with a patient suffering from depression, the possibility of offering psychotherapy rather than medical therapy should be considered, and in the latter case, the impact of the specific drug being considered on dementia risk should be reviewed before it is prescribed.

The literature describes a number of physiological impacts attributed to the use of antidepressants.

### On inflammation

Findings suggest that some antidepressants possess significant anti-inflammatory properties (Tynan et al., 2012; Walker, 2013; Jeon and Kim, 2017). Along with their impact on the cells of the peripheral immune system, selective serotonin reuptake inhibitors (SSRIs) can limit microglial and astroglial inflammatory processes (Dafsari and Jessen, 2020). As an example, fluoxetine causes the downregulation of genes involved in the pro-inflammatory response pathways such as the activation of IL-6 signaling and nuclear factor kappa b (NF-κb) signaling, and of TNFα signaling-related molecules (Patrício et al., 2015). Further, the dopamine enhancer bupropion inhibits pro-inflammatory cytokine production and lowers production of TNFα and interferon γ in mice (Brustolim et al., 2006). Researchers have shown that SSRIs (e.g., sertraline, fluoxetine, and paroxetine) likely inhibit microglial TNFα and nitrous oxide production. In mixed glial cell cultures, serotonin, and norepinephrine reuptake inhibitors (SNRIs) such as the MAO inhibitor moclobemide and selective noradrenaline reuptake inhibitors are anti-inflammatory (Vollmar et al., 2008; Bielecka et al., 2010). The reduction in neuroinflammation resulting from the noradrenaline reuptake inhibitor was also able to partially restore microglial function (Heneka et al., 2015). Popa-Wagner et al. (2014) suggested in 2014 that inflammation may be one pathophysiologic mechanism that contributes to treatment resistance in depression.

The effects of antidepressant treatment on microglial activation in patients with MDD were studied by using 18F-FEPPA PET. It revealed that the longer patients went without treatment, the greater the microglial activation was. However, if the patients were given antidepressants, the increase in microglial activation was no longer observed (Setiawan et al., 2018). The anticholinergic effects of some tricyclic antidepressant drugs have been shown to raise the risk of dementia possibly through accelerated glial transition to a neurodegenerative phenotype (Gamage et al., 2020).

Thus, there is a clear link for both depression and dementia to the inflammatory process, and the ability of any antidepressant approach or treatment to moderate the inflammatory load may be key to its success in reducing dementia.

Other physiological impacts of antidepressants that have been reported to date include.

### On cerebral hemodynamics

Bench et al. (1995) performed early scans of patients suffering from depression and then rescanned the same patients following treatment with an antidepressant medication. They found that recovery from depression was linked to increases in rCBF flow in the same areas in which focal decreases in this parameter were described in the depressed state compared with normal subjects. Similar findings in another study described patients with depression having reduced blood flow to the left frontal brain region. However, with the antidepressant medication venlafaxine, the blood flow was restored (Navarro et al., 2004). Ishizaki et al. (2008) showed that following pharmacotherapy rCBF improved remarkably in the left dorsolateral medial prefrontal cortex (PFC) and the right parietooccipital regions while decreased CBF in some other regions of the PFC did not significantly improve. In a sample of older patients, Wei et al. (2018) reported that rCBF increases were linked to reductions in depressive symptoms. This led the authors to state that their observations were consistent with the vascular depression hypothesis in late-life depression.

### On incidence of dementia

Antidepressant treatment may reduce cognitive decline (Mossello et al., 2008). It is estimated that the incidence of dementia would decline by 4% in the population if antidepressant treatment is applied (Mossello et al., 2008). Bartels et al. (2020) sought to determine the result of antidepressant drug classes on the risk for developing dementia using multiple treatment intervals. The researchers analyzed data of 62,317 individuals with an incident dementia diagnosis who were included in the German Disease Analyzer database and compared outcomes to those of controls matched by age and sex. They conducted logistic regression analyses, which were adjusted for health insurance status and comorbid diseases linked to dementia or antidepressant treatment, to evaluate the links between dementia incidence and treatment with four major classes of antidepressant drug, as well as 14 of the most commonly prescribed individual antidepressants. Results showed an association between treatment for 2 years or longer with any antidepressant and a lower risk for dementia among 17 of 18 comparisons. Particularly for long-term treatment, tricyclic antidepressants were linked to a reduction in the incidence of dementia. Long-term treatment with escitalopram (OR = 0.66; 95% CI, 0.5–0.89) was associated with the lowest risk for dementia on an individual antidepressant basis.

However, it is important to emphasize that antidepressant medications have a host of risks, contradictions and side effects that must be considered for each individual prior to starting treatment. As has been stated, some antidepressants may be ineffective or even have negative effects on cognition (Lu and Tune, 2003; Wang et al., 2016; Moraros et al., 2017).

## Summary and conclusion

This review highlights that depression is a risk factor for dementia and details the physiological steps that link depression to its negative cognitive function. These steps begin with activation of inflammatory mediators, followed by a decline in the density of cerebral small vessels, which then leads to a drop in cerebral blood flow. This sequence is evident in the brains of depressed individuals when they are still cognitively normal but predicts the eventual decline in memory function.

The multifaceted physiological consequences of depression described here conform to the already outlined pattern for other recognized risks for dementia such as obesity, sedentary lifestyle and inadequate sleep (Hakim, 2021). The major difference between depression and the other cognitive risk factors is the possibility of offering various therapeutic modalities to the affected individuals.

This review cautions, however, that some antidepressant medications may worsen the cognitive impact of depression and recommends a careful evaluation of the proposed therapy on the subsequent decline in cognitive function. In addition, the use of social supports to reduce cognitive decline and depression should also be considered. Reducing social isolation has been shown to potentially delay the onset of dementia (Xiang et al., 2021). Positive social support was shown to reduce the risk of dementia whereas negative support increased the risk among persons aged 50 years and over (Khondoker et al., 2017). Overall, high quality social relationships appear to be important for overall cognitive health and can also reduce depression in older people (Murata et al., 2017).

With the identification of the proposed intermediary steps to link depression and other risk factors to cognitive decline, research can focus on identifying and neutralizing the inflammatory mediators, with the goal of interrupting the negative impact they have on cognitive function.

## Author contributions

AH reviewed the literature, wrote the manuscript, and approved the submitted version.
